# Estrogen receptor beta expression in prostate adenocarcinoma

**DOI:** 10.1186/1746-1596-6-61

**Published:** 2011-07-06

**Authors:** Mojgan Asgari, Arman Morakabati

**Affiliations:** 1 Oncopathology Research Center, Tehran University of Medical Sciences (TUMS), Tehran 141555983, Iran; 2 Hasheminejad Clinical Research Developing Center (HCRDC), Tehran University of Medical Sciences (TUMS), Tehran 1969714717, Iran; 3 Ghom University of Medical Science, Ghom 3713649373, Iran

## Abstract

**Background:**

Prostate cancer is the most commonly diagnosed cancer in men and the second leading cause of cancer death in men. Estrogen induction of cell proliferation is a crucial step in carcinogenesis of gynecologic target tissues, and there are many studies recently done, showing that prostate cancer growth is also influenced by estrogen. The characterization of estrogen receptor beta (ER-b) brought new insight into the mechanisms underlying estrogen signalling. In the present study, we investigated the expression of estrogen receptor-b (ER-b) in human prostate cancer tissues.

**Methods:**

We selected 52 paraffin-embedded blocks of prostate needle biopsies in a cross-sectional study to determine frequency and rate of ER-b expression in different grades of prostate adenocarcinoma according to Gleason grading system. Immunohistochemical staining of tissue sections by monoclonal anti ER-b antibody was performed using an Envision method visualising system.

**Results:**

ER-b expression was seen in tumoral cells of prostatic carcinoma in all 29 cases with low and intermediate tumors (100%) and 19 of 23 cases with high grade tumor (83%). Mean rate of ER-b expression in low & intermediate grade cancers was 68.41% (SD = 25.63) whereas high grade cancers showed 49.48% rate of expression (SD = 28.79).

**Conclusions:**

ER-b expression is reduced in high grade prostate cancers compared to low & intermediate grade ones (*P *value 0.027).

## Background

Prostate cancer is the most common non-cutaneous cancer among males and account for 10% of male cancer deaths. Etiology of prostate cancer is mainly unclear but seems to be multifactorial in origin such as alteration of genes, diet, race and hormones [1-3]. Increasing age, weight, and a fat-rich diet as potential risk factors for prostate cancer may be associated with an increase in estrogen levels or high estrogen/androgen levels in circulating blood [4]. Both epidemiological and experimental data suggest that estrogens are involved in prostatic cancer carcinogenesis and tumor progression [5]. Also since the pioneering work of Huggins and co-workers, estrogens have been widely used in the medical treatment of hormone refractory prostate cancer [6]. The role of estrogen in prostate cancer progression is not well understood. It is generally accepted that estrogens have influence on prostatic growth indirectly through effects at the hypothalamic and pituitary levels, reducing gonadotrophin secretion and hence the synthesis of testicular testosterone, but effects of estrogens on target tissues are now known to be mediated by ligand-specific receptor proteins termed estrogen receptor-a and -b (ER-a and ER-b). In prostate, ER-b is preferentially expressed in epithelial cells but ER-a is found in stromal and basal cells [7]. There are evidences of increased ER staining intensity in malignant prostate [8]. ER-a and ER-b belong to the nuclear hormone receptor family, many of whose members are ligand-activated transcription factors that regulate gene expression in a cell- and promoter-specific manner. Expression of estrogen receptors has been found in many other hormone-dependent tumors such as uterus, breast, and also identified in lung cancer and a few brain tumors [9,10]. Most of the physiologic effects of estrogen are attributed to its activity at the ER-a receptor. Although ER-a and ER-b have similar ligand-binding domains and both bind estrogen receptor, there are evidences that ER-a and ER-b demonstrate distinct and sometimes opposing transcriptional activities [11]. All the ERs are widely distributed. ER-a receptors are found in endometrium, breast, ovary and hypothalamus; whereas ER-b has been documented in kidney, brain, skeleton, prostate and endothelial cells [12].

Previous studies using different methodologies have provided evidences that ERs are present in various proportions in normal prostate, prostatic cancer and dysplasia [12]. The role of ER-b in pathogenesis or prognosis of prostate cancer is unclear [4]. It seems to have a role in the control of proliferation and the prevention of hyperplasia in the rodent prostate, as ERb knockout mice show prostatic hyperplasia in aging process. However, the regulatory impact of estrogens on normal and abnormal prostatic growth still remains a matter of great speculations [13].

In this study we examined estrogen receptor -b expression in prostate adenocarcinoma by immunohistochemichal study to determine its frequency and rate of expression in different grades of tumor, defined by Gleason grading system.

## Methods

52 Paraffin-embedded blocks of transrectal ultrasound guided prostate needle biopsies diagnosed as prostate carcinoma and related H&E (Haematoxylin and Eosin) slides and pathology reports retrieved from archive of pathology of "Hasheminejad Kidney Center". All the 52 specimens were judged by the pathologist to contain sufficient tissue for immunohistochemichal analysis. None of the patients had received any hormonal manipulation before the needle biopsy. Tissue sections were cut 4 to 6 μm thick, stained by routine hematoxylin and eosin, and reviewed by pathologist to confirm the diagnosis and re-assign histologic grade according to Gleason grading system criteria. The tumors categorized as "low grade" if Gleason score was equal to or less than 4 and "high grade" if Gleason score was equal to or more than 8. Gleason score of 5, 6 and 7 was considered as intermediate grade. We considered low and intermediate grade as one group if the score was less than 8. For immunohistochemichal staining, tissue sections were deparaffinised, and rehydrated. Heat-induced epitope retrieval was achieved by boiling sections in the EDTA buffer at pH 8.9 in a high-pressure cooker for 20 minutes (4 × 5 minutes). Sections were allowed to cool at room temperature, rinsed thoroughly with water, and placed in Tris-buffered saline. Sections were incubated for 5 minutes with peroxidase block solution (Envision; Dako, Glostrup, Denmark) to neutralize endogenous peroxidases. Then sections were incubated for 30 minutes at room temperature with mouse anti-human ER-b; clone PPG5/10 (1:2 dilution; Serotec, Oxford, UK). Immunostaining was performed using the Envision method (Dako) according to the manufacturer's instructions. Appropriate positive and negative controls were used. Estrogen receptor-b expression thoroughly sought for in tumoral cells as any nuclear staining seen in tumoral cells. Etrogen receptor-b expression assigned as positive when more than 10% of tumoral nuclei were stained. Rate of ER-b expression defined as percentage of positive nuclei per 200 cells counted.

Statistical analysis was performed using SPSS 13.0 software. Chi square and Mann-Whitney U test were used for comparative analysis.

## Results

**Figure 1:**
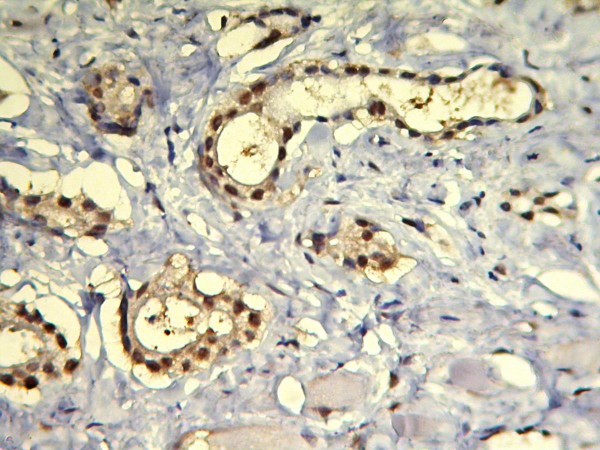
**Prostate carcinoma Gleason score 3+3 showing > 70% positive nuclei stained by IHC for ER-b**.

**Figure 2:**
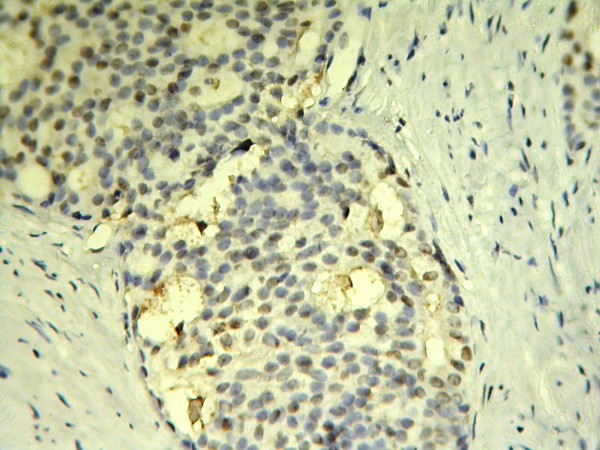
**Cribriform architecture in a Gleason score 4+4 prostate carcinoma with negative immune reaction**.

A total of 52 cases of prostatic adenocarcinoma were studied including 29 low and intermediate grade cancers and 23 high grade cancers. Mean age of patients was 67 ± 8 (range:55-84). Using immunohistochemichal method and 10% nuclear staining as the cut off point, 48 prostate adenocarcinoma expressed ER-b (92.3%) and 4 cases were ER-b negative (7.7%) which all of them were high grade cancers (Table 1). We found ER-b expression in tumoral cells of prostate carcinoma in 29 of our low and intermediate grade cancers (100%) and 19 of high grade cancers (83%) but 4 high grade cancers were negative (17%). Considering percent of positive nuclei counted in 200 cells, the mean rate of ER-b expression in low & intermediate grade cancers was 68.41% (SD = 25.63) whereas high grade cancers showed 49.48% rate of expression (SD = 28.79). (Figure 1, 2)

**Table 1:** Frequency of ER-b expression based on Gleason score and tumor grade

	Low & intermediate grade		high grade			total
**Gleason grade**	6	7	8	9	10	
**ER-b (+)**	15	14	4	11	4	48
**ER-b (-)**	0	0	2	2	0	4
**Total**	15	14	6	13	4	52

## Discussion

We found ER-b expression in all of our low and intermediate grade cancers and in 83% of high grade cancers but 17% of high grade cancers were negative (*P *value 0.019). Our study reveals reduced expression of ER-b in higher grade prostatic adenocarcinoma compared to low & intermediate grade ones (*P *value 0.027).

In this study, we used immunohistochemichal staining on paraffin-embedded blocks of prostate needle biopsy for determining ER-b expression. Using prostate needle biopsy specimen for immunohistochemichal studies of prostate carcinoma has both advantages and disadvantages. Considering the uniform thickness of specimens in prostatic needle biopsies, tissue fixation is more rapid and even in compare with prostatectomy specimens. However, the small size of the specimen has some limitations, such as missing focal small lesions in recut sections. An additional advantage of primary diagnostic needle biopsy is that the patients usually have not received any treatment before the biopsy.

Our data supports that rate of ER-b expression is significantly lower in high grade tumors than in low & intermediate ones (*P *value 0.027). These findings are agreed with most of currently reported articles.

Leav.et al, using a different monoclonal anti ER-b antibody (anti GC-17), showed ER-b staining present in the majority of grade 3 carcinomas of the peripheral zone but greatly diminished or absent in most grade 4/5 carcinomas [14].

Lisa G. Horvath et al studied patterns of ER-b expression in normal, hyperplastic, and prostate carcinoma using a different primary antibody (chicken polyclonal antibody (ER-b 503 IgY3) for immuonohistochemistry. In their study; all normal prostates showed strong ER-b nuclear staining in > 95% of the epithelium and 35% of the stromal cells. The number of ER-b positive cases declined to 24.2% (38/157) in prostatic hyperplasia adjacent to carcinoma and 11.3% (18/159) in prostate cancers. They conclude that ER-b is highly expressed in normal human prostate and there is a progressive loss of expression in prostatic hyperplasia and, to a greater extent, in invasive cancer [15].

On the other hand, Bonkhoff et al, have tested two commercially available antibodies in routinely processed and frozen prostate carcinoma tissue sections. The 65-kd anti-rat estrogen receptor b (Upstate Biotechnology, Lake Placid, NY) and 210-180-C050 antibody (Alexis Corporation, Nottingham, UK). They showed that low and intermediate grade adenocarcinoma expressed ER-b in a minority of cases and high grade (primary Gleason grades 4 and 5) cancers revealed at least focal ER positivity in 43% (respectively 61%) of cases [4].

In 2003 Fixemer T. et al use a different monoclonal antibody in immuonohistochemistry, reports higher ER-b expression in cancer with Gleason grade IV than in cancers with grades III and V and suggest that ER-b protein expression decreases during cancer progression, but no correlation was found between the ER-b status and the primary Gleason grade [16].

It seems there is no obvious explanation for these controversies between different reported results on the levels of ER-b expression. It is well known that imperfect antibody specificity or different primary antibodies, ineffective antigen retrieval and tissue-processing methods, or the presence of unknown isoforms of ER protein may affect immuonohistochemistry performance. A possible implication of ER-b in neoplastic growth control is suggested by the findings of a selective loss of ER-b protein in colon adenocarcinoma and ovarian cancer [17]. ER-b seems to have a role in the control of proliferation and the prevention of hyperplasia in the rodent prostate, as ER-b knockout mice show prostatic hyperplasia on aging [13].

## Conclusions

In summary we investigated ER-b expression in prostatic adenocarcinoma with different grades and we found reduced expression of ER-b in higher grade prostatic adenocarcinoma compared to low & intermediate grade ones. It seems more studies on influence of ER-b expression on prostate cancer staging and other prognostic factor would be more helpful to find the role of ER-b in human prostate cancer. This observation may have clinical implications as tumor cell expressing these protein are potentially estrogen responsive and will survive in an androgen-deprived situation and also if there is any need for treating the cancer with anti-estrogens or not.

## Competing interests

The authors declare that they have no competing interests.

## Authors' contributions

This work is a thesis of AM that was done under supervision of MA. AM selected the cases for IHC. IHC slides reviewed by AM and MA. Both wrote, read and approved the final manuscript.
